# An evaluation of a model for the systematic documentation of hospital based health promotion activities: results from a multicentre study

**DOI:** 10.1186/1472-6963-7-145

**Published:** 2007-09-18

**Authors:** Hanne Tønnesen, Mette E Christensen, Oliver Groene, Ann O'Riordan, Fabrizio Simonelli, Lagle Suurorg, Denise Morris, Peder Vibe, Susan Himel, Poul Erik Hansen

**Affiliations:** 1 Clinical Unit of Health Promotion/WHO Collaborating Centre for Evidence Based Health Promotion in Hospitals, Bispebjerg Hospital, DK 2400 Copenhagen, Denmark; 2 WHO European Office for Integrated Health Care Services, E 08006 Barcelona, Spain; 3 James Connolly Memorial Hospital, Blanchardstown, IR Dublin 15, Ireland; 4 A. Meyer Children's University Hospital, I 50132 Florence, Italy; 5 National Institute for Health Development, EE 11619 Tallinn, Estonia; 6 Lancashire Teaching Hospitals Foundation Trust, UK PR2 9HT Preston, UK; 7 Orthopedic Department, Hässleholm Hospital, S 28125 Hässleholm Kristianstad, Sweden; 8 Trillium Health Centre, Mississauga, ON L5B 1B8, Canada; 9 Danish National Board of Health, DK 2300 Copenhagen, Denmark

## Abstract

**Background:**

The first step of handling health promotion (HP) in Diagnosis Related Groups (DRGs) is a systematic documentation and registration of the activities in the medical records. So far the possibility and tradition for systematic registration of clinical HP activities in the medical records and in patient administrative systems have been sparse. Therefore, the activities are mostly invisible in the registers of hospital services as well as in budgets and balances.

A simple model has been described to structure the registration of the HP procedures performed by the clinical staff. The model consists of two parts; first part includes motivational counselling (7 codes) and the second part comprehends intervention, rehabilitation and after treatment (8 codes).

The objective was to evaluate in an international study the usefulness, applicability and sufficiency of a simple model for the systematic registration of clinical HP procedures in day life.

**Methods:**

The multi centre project was carried out in 19 departments/hospitals in 6 countries in a clinical setup. The study consisted of three parts in accordance with the objectives.

A: Individual test. 20 consecutive medical records from each participating department/hospital were coded by the (coding) specialists at local department/hospital, exclusively (n = 5,529 of 5,700 possible tests in total).

B: Common test. 14 standardized medical records were coded by all the specialists from 17 departments/hospitals, who returned 3,046 of 3,570 tests.

C: Specialist evaluation. The specialists from the 19 departments/hospitals evaluated if the codes were useful, applicable and sufficient for the registration in their own department/hospital (239 of 285).

**Results:**

A: In 97 to100% of the local patient pathways the specialists were able to evaluate if there was documentation of HP activities in the medical record to be coded.

B: Inter rater reliability on the use of the codes were 93% (57 to 100%) and 71% (31 to 100%), respectively.

C: The majority of the study participants found the codes to be useful (71%), applicable (92%) and sufficient (92%).

**Conclusion:**

Systematic registration of HP activities is relevant in clinical day life and the suggested codes proved to be applicable for international use. HP is an essential part of the clinical pathway or the value chain. This model promises to improve the documentation and thereby facilitate analysis of records for evidence based medicine as well as cost and policy analyses.

## Background

There is increasing evidence on the effectiveness of clinical health promotion (HP) services, which include disease prevention, HP and rehabilitation services and aim at actively involving patients in the care process [1,2]. Several evidence based guidelines and programs have been described to significantly reduce morbidity and mortality and improve recovery, treatment outcomes and prognosis. Concrete examples of evidence based clinical HP services are for example preoperative smoking cessation and alcohol intervention for patients undergoing elective surgery [3,4], early rehabilitation after stroke [5], integrated rehabilitation program for diabetic patients [6], after treatment program for children suffering from asthma [7], patient education for patients suffering from chronic diseases [8]. The effects of clinical HP services can be substantial and include reduced morbidity, complications, second surgery, rehospitalisation or death, as well as intermittent outcomes, such as higher patient satisfaction, improved lifestyle, shorter hospital stay, and lower costs.

The changing patterns of diseases require hospital services to integrate evidence based clinical HP as a natural element in the patient pathways. This cannot be achieved through revised clinical guidelines or quality standards alone, but requires a change in the way services are purchased.

A number of internationally accepted coding systems for hospitals exists. The most well known belong to the family of the International Classification of Disease (ICD), such as ICD 9, ICD 9 CM and ICD 10 [9]. The current version is the ICD 10, which introduced in 1992.

The ICD 9 was published in 1977, and this version is still being used in several countries [10]. ICD 9 CM is the clinical modification of the ICD 9 codes developed by the National Centre for Health Statistics (NCHS) and the Centres for Medicare and Medicaid Services in the United States. ICD 9 has 6,969 codes while there are 12,420 codes in ICD 10, 14,199 with the fourth character place of occurrence codes in the new chapter XX: External Causes of Morbidity and Mortality [11]. ICD 9 CM also includes codes for procedures, which are not included in either ICD 9 or ICD 10. However, none of the existing ICD systems allows for the coding of health promotion activities.

The latest classification system International Classification of Functioning, Disability and Health (ICF) includes more than 100,000 codes, which give the possibility of describing the status of the functioning, disability and health as well as the resources and barriers of the patient and the society in a very detailed bio/psycho/social model [12]. However, at present ICF is complicated and time consuming when used for ordinary somatic patient pathways. It does not include the most common HP activities and the clinical use is therefore often restricted to minor groups of patients with multiple handicaps requiring long term rehabilitation and revalidation from several sectors and specialists.

The Diagnosis Related Groups (DRGs) system for reimbursement was developed in the nineteen seventies to control health care costs and today the DRG is implemented widely. Most reimbursement is related to diagnoses, except for surgery where the activity is registered, and the major part of the reimbursement is related to this activity.

The first step of handling clinical HP services in the DRGs is a systematic documentation and registration of the activities in the medical records. A Danish survey had showed that less than 10% of the patient related HP activities taking place in hospitals were registered in the National Patient Registry [13,14].

There is in fact a dearth of tradition for documentation of clinical HP activities in the medical records and international and national classifications as well as other patient administrative systems include only few and often non systematic registration codes to cover these activities. As a result of the discrepancy between delivering and registering clinical HP in hospitals the services are nearly invisible in budgets and balances, registration of procedures and diagnoses, quality management and in clinical databases of outcome. Therefore it is difficult to prioritize resources for clinical HP and to assess the effect of such interventions in terms of outcomes and costs.

To overcome these problems it is necessary to develop a comprehensive, but simple model for the systematic registration of the most important and frequent clinical HP activities that is compatible with current patient administration systems. In order to be meaningful to clinicians the model should be related to the patient pathway, thus including the motivational counselling as well as subsequent intervention programs. It should cover risk factor related as well as integrated rehabilitation programs.

A first generation model was developed in Denmark, pilot tested nation wide, and adjusted and integrated in the Danish Classification System (table 1) [15]. This system includes the International Classification of Diseases (ICD)[9], Nordic Classification of Surgical Procedures (NCSP)[16], International Classification of Functioning, Disability and Health (ICF) [12], and others.

**Table 1:** New comprehensive model for registration of clinical health promotion activities aimed hospital patients.

**7 codes for motivational counselling and motivational interviewing technique related to:**	
Tobacco	XX01
Alcohol	XX02
Nutrition	XX03
Physical activity	XX04
Psycho social relations	XX05
Other risk factors	XX06
	
Integrated counselling	XX07

**8 codes for intervention, rehabilitation and after treatment, including:**	

Smoking cessation program	YY01
Alcohol intervention program	YY02
Nutrition program	YY03
Physical exercise intervention	YY04
Psycho social support	YY05
Medical optimization	YY06
Patient education program	YY07
	
Integrated rehabilitation (consisting of several factors)	YY08

We tested the registration model for clinical HP in an international study in order to assess whether it is applicable to real life situations in other countries and in order to evaluate its usefulness, applicability and sufficiency.

## Methods

The model included registration codes for the (initial) concrete motivational counselling and interviewing technique as well as the (following) regular intervention, rehabilitation and other treatment programs. Diagnoses, giving out flyers, recommending or referring to intervention were not defined as clinical health promoting activities and they are therefore not included in the model. This registration corresponded to the well established registration of surgery: only operations performed are registered as surgery.

This multicentre project involved 19 departments in 6 countries (Canada, Estonia, Ireland, Italy, Sweden, United Kingdom) in a clinical setting. The departments included a children's hospital and hospital departments of surgery, orthopaedic surgery, internal medicine and geriatrics, cardiovascular disease, and psychiatry from university hospitals as well as other hospitals. The clinical specialists in all but three departments carried out the project. The last had a registration routine, which included a group of coding specialists without physicians.

In order to perform the evaluation under normal clinical circumstances, the evaluation took place in the clinical setting routinely used for registration and coding at each of the 19 departments. A coordinator from each country or region was responsible for driving the process and delivering the results within the deadlines. No patients were contacted directly or indirectly.

According to the objectives, the project consisted of three parts. Since the tradition for registration of health promoting activities has been only sparse, it was necessary that the specialists should get used to the codes in part A and part B before they could assess the codes in terms of usefulness, applicability and sufficiency.

### A) Individual test in local conditions

In order to reflect the clinical setting at each department, this first part was based on local material and registration routines, thus using local medical records (in local language). The specialist collected and coded 20 consecutive records from his or her own department. The medical records were taken consecutively in one or more following days, according to the local routine procedure until the number of 20 was reached. The specialist then checked the records for documentation of clinical HP activities in the registration model. The criteria for coding were as follows:

For each activity there were two categories, "yes" and "no". The specialist should answer "yes", if the activity was performed according to written proof in the medical record, otherwise the answer should be "no".

A medical record was defined as data obtained from the records or documentation maintained on a patient in any health care setting (for example, hospital, home care, long term care, practitioner office). It included automated and paper medical record systems, medication profiles, nursing care plans and other written material. The data were collected on separate registration forms with identification numbers for the specialist and the department, but not for the patients. Though it was beyond the scope of the study, the numbers of activities performed at each department were also reported. All 19 departments responded.

The numbers of tests in part A) were 20 medical records × 19 departments × 15 codes = 5,700 tests in total. The responders performed 5,529 tests (= 97%).

### B) Common test of international conditions

In order to control the test material coding procedure and to compare the coding results and the agreement among the specialists, the next step was an evaluation using standardized medical records in all departments. Therefore, 14 medical records were selected, translated into English and distributed by the Collaborating Centre to all the specialists, of whom 17 responded (response rate: was 89%). The responders performed 3,046 of (14 × 17 × 15 =) 3,570 tests.

Agreement on the use of codes was obtained, when the majority (meaning more than half) and the qualified majority (defined as more than two thirds), respectively, of the specialists had used the same code.

The analyses for agreement included 136 for each registration code (see table 2). The number originated from 17 departments × 17 departments = 289; excluding " the possibility for agreement with your self" 289 - 17 departments = 272; using one sided evaluation 272/2 = 136.

**Table 2:** Working table showing the details of the calculation of agreement for yy04.

YY04	H/D^1^	H/D^2^	H/D^3^	H/D^4^	H/D^5^	H/D^6^	H/D^7^	H/D^8^	H/D^9^	H/D^10^	H/D^11^	H/D^12^	H/D^13^	H/D^14^	H/D^15^	H/D^16^	H/D^17^
H/D^1^	-----																
H/D^2^	86%	-----															
H/D^3^	93%	93%	-----														
H/D^4^	79%	79%	71%	-----													
H/D^5^	86%	86%	79%	79%	-----												
H/D^6^	**50%**	**#64%**	**#57%**	71%	**#50%**	-----											
H/D^7^	79%	**#64%**	71%	**#57%**	79%	**29%**	-----										
H/D^8^	71%	71%	**#64%**	79%	86%	**50%**	**#64%**	-----									
H/D^9^	93%	93%	86%	86%	93%	**#57%**	71%	79%	-----								
H/D^10^	79%	93%	86%	86%	79%	71%	**#57%**	79%	86%	-----							
H/D^11^	79%	79%	71%	86%	79%	71%	**#57%**	79%	86%	86%	-----						
H/D^12^	79%	79%	71%	71%	93%	**43%**	71%	93%	86%	71%	79%	-----					
H/D^13^	79%	93%	86%	71%	79%	71%	**#57%**	**#64%**	86%	86%	71%	71%	-----				
H/D^14^	100%	86%	93%	79%	86%	**50%**	79%	71%	93%	79%	86%	79%	79%	-----			
H/D^15^	79%	79%	71%	86%	79%	71%	**#57%**	79%	86%	86%	79%	71%	86%	79%	-----		
H/D^16^	79%	79%	71%	86%	79%	71%	**#57%**	79%	86%	86%	100%	71%	86%	79%	100%	-----	
H/D^17^	100%	86%	93%	79%	86%	**50%**	**50%**	71%	93%	79%	79%	79%	79%	100%	79%	79%	-----
**>50%**	**15**	**15**	**14**	**13**	**11**	**6**	**9**	**9**	**8**	**7**	**6**	**5**	**4**	**3**	**2**	**1**	**128**
**#>66%**	**#15**	**#13**	**#12**	**#12**	**#11**	**#5**	**#3**	**#8**	**#8**	**#7**	**#6**	**#5**	**#4**	**#3**	**#2**	**#1**	**#115**

> 50%: 128 of 136 = 94% # > 66%: 115 of 136 = 85%

### C) Specialist evaluation

All specialists from the 19 departments/hospitals evaluated if the individual codes were useful, applicable, and sufficient for the registration in their own department.

Useful was defined as whether the code was useful in daily clinical life. Applicable was defined as whether the code was applicable to the registration procedure in the individual department. Sufficient was defined as whether the code was sufficient for the patient groups and the activity it covered.

The numbers of evaluations were 19 × 15 = 285 in total. The specialists performed 239 evaluations (= 84%).

### Statistical analysis and Ethical considerations

The results are given in absolute numbers, frequencies, or median and range. Kappa statistic was calculated to assess the agreement in registration among the specialists in part B (interobserver variation) [17]. Kappa was not weighted or adjusted, because all patient pathways were open for assessment of agreement. A kappa value ranging from 0.41 to 0.60 indicates moderate agreement, 0.61 to 0.80 substantial agreement, and 0.81 to 1.0 near perfect agreement [18].

The data collection and report were chosen to be completely anonymous. The evaluation did not include ethical problems, since no patients were directly involved or contacted, and since all data were anonymised before collecting. According to Danish Research Policy, since the registration only concerned doctors and organisations, it was not necessary to seek patient consent. The study has been approved by the Ethical Committee at Bispebjerg University Hospital.

## Results

### A) Individual test in local conditions

The specialists were able to categorize if the HP activities were documented of in the medical record or not in 97 to100% of the local cases (figure 1, closed bars).

Furthermore the frequencies of HP activities in the individual departments were median 29,6 (range 10,3 to 36,2) for motivational activities and 29,4 (22,0 to 40,1) for intervention, rehabilitation and after treatment (figure 1, streaked bars).

### B) Common test of international conditions

The agreement among the specialists when coding the same medical records was 93% (57 to 100%) when using simple majority, and 71% (31 to 100%) when using the qualified majority (see figure 2 and table 2). The Kappa was correspondingly high ranging from 0.61 to 0.93, interpreted as ranging from substantial to almost perfect agreement.

### C) Specialist evaluation

The majority of the specialists found the codes to be useful (71%), applicable (92%) and sufficient (92%) in the daily life.

The specialists had few comments, mostly regarding the unsystematic documentation of clinical HP activities in the medical records. Several asked for a simple systematic identification of clinical relevant risk factors, which would often precede the HP activity. Other comments concerned the minor groups of their patients or diagnoses, who/which were not or only partly covered by the registration codes; especially cancer patients in terminal stage and parents to hospitalised children undergoing education programs.

## Discussion

We found that the model for systematic registration of clinical HP was useful, applicable, and sufficient in a clinical setting in 19 hospital departments in six countries. The response rate and the agreement were relatively high. The coding specialists had only few comments regarding the registration. They predominantly focused upon their weak documentation in the medical records, and asked for a simple tool for systematic identification of clinical relevant risk factors.

These international results were surprisingly positive, which could be due to several factors such as a special interest in clinical HP activities or DRGs among the participating specialists and their management. The results may have been different in other settings or using other methods. However, as the results are in agreement with the results from a previous national pilot test they may reflect consensus on the model for registration of HP activities in clinical settings. The number of departments and hospitals was too small to make international comparisons or comparisons on specialities.

The registration model represents HP activities instead of diagnoses, because for instance the diagnosing of smoking, overweight, or diabetes is not automatically followed by an offer of the relevant and evidence based HP activities. This is in contrast to hospital routines, where a diagnosis of thrombosis is followed by anti thrombosis treatment, the appendicitis by an appendectomy, and the pneumonia by the relevant antibiotics. Because of the incomplete clinical decision making in the field of clinical HP it seems most relevant to focus on these activities (including for reimbursement systems), until new traditions are implemented.

The method used in this project was chosen in order to reflect clinical day life. When testing the model on local consecutive material as well as international material it showed the general problem, that there are no standardised designs for research concerning coding of activities. Furthermore the available statistical methods are often weak and without defined level of significance, which is the case for the kappa statistic as well as for other statistical analyses, which could be relevant to use in future studies in this field [17]. We have therefore described the methods used in the present study as transparent as possible.

We could have used other methods, such as focus interviews in the present study, but this would have reflected the attitude of the specialists rather than the clinical routines, and the evidence level would not have increased [19]. Based on the results from this study, we hope that other researchers will repeat the evaluation in other settings or develop methodologies for larger scale research on the use of codes for the registration of clinical HP activities. Inspiration could be found in the research concerning quality of diagnosing and coding [20].

The model presented in this article fulfils some of the requirements for systematic registration of clinical HP activities relevant for clinical departments, which are not included in the current classification systems.

Implementing the new coding model may be limited by several factors. The medical doctors and others responsible for the coding procedures need to introduce new traditions of documentation and registration of clinical HP activities in order to fullfil the obligation for documentation of health services and patient related activities, inclusive HP activities. Applying the model presented in this paper in clinical practice may increase resources for coding, however, according to the international standard operation procedures the codes would have to be placed only, if an activity takes place and is documented in the medical record, therefore the additional time spent on registration is very limited. According to coding in DRGs the HP codes may also be connected to over coding and creep (deliberate or inadvertent misclassifications) [21]. A recent study has shown that payment in primary care for identifying and referring patients with tobacco use disorder only resulted in improved diagnosing, not more cessation programs [22]. Unfortunately the payment was not given for performing smoking cessation interventions (motivational interviewing technique or regular cessation programs). The results are not contradictory to an incitement strategy that reimburses the clinical health promoting activity performed instead of diagnosing, recommending and referring.

The focus of this research was on the hospitals' role to clinical health promotion exclusively. Primary care has made very important contributions, but was not part of the project and does not affect the generalizability of the results among the hospitals.

The work on developing the model presented in this article was coordinated with a working group developing standards and indicators for Health Promotion in Hospitals [23]. As a future application of our work, the HP codes could be used directly to facilitate the construction of clinical health promotion indicators and thus included in the hospitals' quality management system. In practice it will be easier to monitor the clinical HP activities in hospitals, either alone by assessing the frequencies of registered activities or by evaluating the services provided to patients with certain diagnoses. It would also be possible to follow up the number of motivational counselling sessions and stop smoking interventions performed among patients with smoking behaviour at department level, or the number of patients suffering from stroke that received the necessary rehabilitation program according to the local or national clinical guidelines. The intervention, rehabilitation and aftertreatment could be registered in details by the components or as an integrated intervention.

The next step should be to implement the clinical HP codes in the national or regional classification systems and to follow up the use. It is recommendable to make the HP codes visible and easy to find among the thousands of other codes. A smart solution would be to include the codes in a new chapter regarding clinical health promotion in the classifications and preserve simplicity and comprehensiveness of this new field of documentation and registration.

The model creates a platform for quality based reimbursement of HP activities, and a following step would be to place prices on the activities through the DRG system. This could be done in several ways using average or individual costs. According to the low costs and the high effectiveness of clinical health promotion in general, it may be more relevant to use an incitement strategy characterised by significant prices for HP activities and/or an extra bonus sum distributed annually.

A WHO workshop on Quality based Reimbursement strategies concluded in 2003 that there were no technical barriers for connecting the clinical HP codes to the national reimbursement systems in Europe and America [24]. A rough estimate for Denmark indicates that the costs for the clinical HP activities would account for less than 1% of the hospital budget usually dedicated for treatment activities. The Danish National Board of Health is working on the visibility of clinical HP activities in their Activity Based Costs Analyses from 2006.

## Conclusion

In conclusion, systematic registration of HP activities is relevant in clinical day life and the first step for handling clinical HP activities in the DRGs has been evaluated in the study presented in this paper. HP is an essential part of the clinical pathway and value chain. Moreover, there is no way that quality can be improved and costs contained without better recordkeeping. This model promises to improve the documentation in the record and thereby facilitate analysis of records for evidence based medicine as well as cost and policy analyses.

## Competing interests

HT is a surgeon as well as director of WHO Collaborating Centre for Evidence Based Health Promotion in Hospitals; is working together with the National Board of Health and the Danish Medical Association; is organising pre and postgraduate education; and acts as peer reviewer on the subject at scientific journals.

The authors MEC, OG, AOR, FS, LS, DM, PV, SH and PEH declare that they have no competing interests.

## Authors' contributions

HT has made substantial contributions to conception and design, acquisition of data, and analysis and interpretation of data. HT has been involved in revising the manuscript critically for important intellectual content and has given final approval of the version to be published.

MEC has made substantial contributions to acquisition of data, been involved in drafting the manuscript and has given final approval of the version to be published.

OG has made substantial contributions to conception and design and analysis and interpretation of data. OG has been involved in revising the manuscript critically for important intellectual content and has given final approval of the version to be published.

PEH has made substantial contributions to conception and design. PEH has been involved in revising the manuscript critically for important intellectual content and has given final approval of the version to be published.

AOR, FS, LS, DM, SH and PV have made substantial contributions to acquisition of data. All have been involved in revising the manuscript critically for important intellectual content and has given final approval of the version to be published.

## Pre-publication history

The pre-publication history for this paper can be accessed here:


